# Wound Care Self-Efficacy Assessment of Italian Registered Nurses and Wound Care Education in Italian Nursing Education System: A Cross-Sectional Study

**DOI:** 10.3390/nursrep12030067

**Published:** 2022-09-18

**Authors:** Nicola Ielapi, Davide Costa, Antonio Peluso, Carmelo Nobile, Veronica Venditti, Egidio Bevacqua, Michele Andreucci, Umberto Marcello Bracale, Raffaele Serra

**Affiliations:** 1 Department of Public Health and Infectious Disease, “Sapienza” University of Rome, 00185 Rome, Italy; 2 Interuniversity Center of Phlebolymphology (CIFL), International Research and Educational Program in Clinical and Experimental Biotechnology, University Magna Graecia of Catanzaro, 88100 Catanzaro, Italy; 3 Department of Law, Economics and Sociology, University Magna Graecia of Catanzaro, 88100 Catanzaro, Italy; 4 Department of Public Health, Vascular Surgery Unit, University Federico II of Naples, 80138 Naples, Italy; 5 Department of Pharmacy and Health and Nutrition Sciences, University of Calabria, 87036 Rende, Italy; 6 Department of Health Sciences, University Magna Graecia of Catanzaro, 88100 Catanzaro, Italy; 7 Department of Medical and Surgical Sciences, University Magna Graecia of Catanzaro, 88100 Catanzaro, Italy

**Keywords:** nurse, education, wound care, wounds, university

## Abstract

Wounds are a major public health challenge for nurses, and poor wound care has important implications for patients and health care systems. The aim of this study is to assess the Italian registered nurses’ (RNs) perception in the area of wound care, regarding their knowledge, tasks of care delivery, wound management, values, and attitudes, exploring also the previous specific education received during nursing education. An observational online web-based survey was used to assess learning goals and content for wound care education in undergraduate nursing education and the skills and level of self-efficacy in this area during clinical practice. The data were collected between April and May 2022. A total of 210 RNs were interviewed and divided into five national geographic areas. Northwestern RNs showed a better education about the wound care area during university courses: the rate of RNs that did not receive any training in the wound care area was lower than in other Italian geographical areas. Southern RNs presented a better knowledge about factors that expose the wound to becoming chronic, wound drains care, and the ability to assess diabetic foot. This study showed that, in Italy, education in wound care among nursing students is relatively poor, and many skills are achieved during an RN’s career in an empirical way.

## 1. Introduction

Wounds (acute or chronic) are a major public health challenge for nurses, because of their high prevalence in the general population. Vascular (arterial, venous, and lymphatic) ulcers, diabetic foot, ulcers, pressure ulcers, and skin tears are the most common types of chronic wounds [1,2,3,4,5,6]. In addition to chronic wounds, nurses deal also with acute wounds such as surgical incisions, as they play a crucial role in wound healing during the recovery and rehabilitation of operated patients to avoid surgical site infections, wound dehiscence, and delayed wound healing of the surgical site [7,8,9]. Moreover, among acute wounds, we have traumatic wounds, burns, and frostbite injuries [10,11,12]. Therefore, wound care is an essential activity of registered nurses’ (RNs) daily practice, and nursing education and training are pivotal for the acquisition of specific wound care skills [13]. Several studies focused on the issue of competence in the wound care area, and they found quite limited competence among RNs and even among graduating nursing students that blame having not received enough education in this area, and undergraduate nursing education is not standardized [14,15,16,17,18]. A recent study [14] assessed learning goals and content for wound care education in a nursing academic program in Finland and showed how the skills related to wound care competence are gained during undergraduate nursing training.

In Italy, following the Bologna process, RNs are educated at universities with a Bachelor’s degree encompassing a three-cycle education (from Bachelor’s to Doctorate degrees). In particular, this includes 3 years of full-time studies (Bachelor’s degree, which is the required step to practice as an RN after the regulative board registration) and, possibly, in addition, 2 years of full-time studies for a Master’s degree in Nursing and Midwifery Sciences (required managerial career), and a further 3 years for a Doctorate course for an academic career. For wound care specialistic education, a postgraduate diploma in wound care (1 year of full-time studies) can be attended after completion of the bachelor’s degree [19,20]. Supplementary Material S1 shows the list of Italian universities providing Bachelor’s degrees in Nursing, Supplementary Material S2 shows the list of Italian universities providing Master’s degrees in nursing and midwifery sciences, and Supplementary Material S3 shows the list of the universities that provide postgraduate diploma in wound care.

In Italy, out of 98 universities (https://www.universitaly.it/index.php/cercacorsi/universita) (accessed on 20 May 2022) [21], 42 universities provide Bachelor’s degrees in nursing, while 34 universities provide Master’s degrees in nursing and midwifery sciences. For both types of degree, therefore, it is possible to observe a sufficient distribution throughout the Italian territory from north to south. As regards, however, specialist wound care training, to date, this accounts for only four universities: two in the northern part and two in the southern part of the Italian territory (Supplementary Material S3). (https://www.almalaurea.it/lau/postlaurea/aa2019-2020) (accessed on 20 May 2022) [22].

The aim of this study is to assess the Italian registered nurses’ (RNs) perception in the area of wound care, regarding their knowledge, tasks of care delivery, wound management, values, and attitudes, referencing also the previous specific education received during nursing education.

## 2. Materials and Methods

### 2.1. Design

We performed an observational online web-based survey that was used to assess learning goals and content for wound care education in undergraduate nursing education.

The study complies with STROBE reporting guidelines for observational research. (https://www.strobe-statement.org/index.php?id=strobe-home, accessed on 5 September 2022) [23].

### 2.2. Setting

The observational online web-based survey used in this study was conducted using Google Modules, structured as in previous works [24,25].

### 2.3. Survey Online Structure

The survey was built based on learning objectives and content for wound care based on areas of expertise as indicated in the papers of Kielo-Viljamaa [13,14,17]. Specifically, we used four competence areas: (1) Anatomy and physiology, (2) Care of chronic and acute wounds, (3) Wound management and assessment, and (4) Values and attitudes. Each learning goal and piece of content was assessed for its clarity, relevance, and importance using a seven-point scale (Likert scales): —strongly disagree 1–2-3–4–5–6–7 strongly agree—. The full questionnaire is included in Supplementary Material S4. The questionnaire was pilot tested with 15 nurses before the onset of the study.

### 2.4. Procedures in Place to Check against Bots Completing the Survey

Although the risk of bots completing the survey was low, as this survey provided no compensation for participants, three open-ended questions (age; educational qualification—“Other Master’s degree” option; actual work—“other” option), located at the beginning of the survey, were used to detect bots and enabled us to check the answers for consistency.

### 2.5. Participants

The participants of the survey had a Bachelor’s degree in Nursing, a Master’s degree in Nursing and Midwifery Sciences, a Postgraduate diploma in wound care, or a combination of the various titles. The current work positions were different: ward nurses, home nurses, private nurses, or other. The selection criterion for the survey was only to be a nurse.

### 2.6. Data Collection

The data were collected between April 2022 and May 2022 using Google Modules, and RNs were interviewed and divided into five different geographic areas, according to the Nomenclature of Statistical Territorial Units of Italy (NUTS: IT, level 1) (https://ec.europa.eu/eurostat/web/nuts/nuts-maps) (accessed on 20 May 2022) [26]. Participants were recruited using different networks in organizations, such as hospitals, via email, telephone, and social networks. Participants were also recruited using social media nursing groups. The open link to the online questionnaire was invited. The recruited participants were invited to use their own social networks to share the link with their colleagues to improve the representativeness of the sample.

### 2.7. Data Analysis

The data from the survey were analyzed using Stata version 16 (StataCorp, College Station, TX, USA). Continuous variables (“age”, “competence area in wound care”, “wound management and care of a patient with a wound”, “values and attitudes”) were analyzed by one way-ANOVA test, after verifying their normal distribution and homoscedasticity using the Shapiro–Wilk normality test and F test, respectively. All continuous variables were also subjected to post hoc analysis using the Tuckey test to show any significant comparison. Categorical variables (“females”, “educational qualification”, “current work”, “educational wound care area”) were analyzed with a five-sample test for the equality of proportions without continuity correction.

## 3. Results

No bots completing the survey were detected and all the answers were considered eligible for analysis.

**Figure 1:**
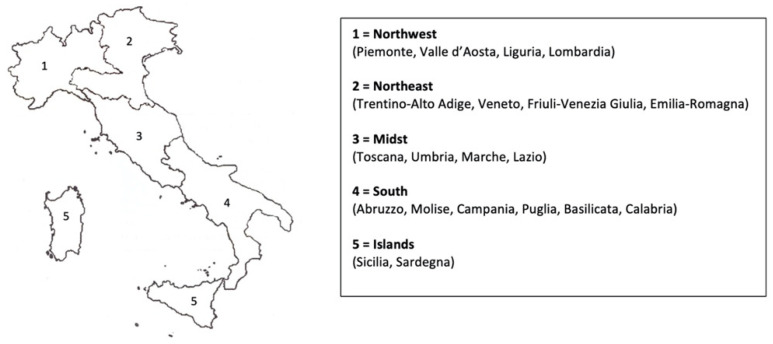
Division of Italian geographical areas according to the Nomenclature of Statistical Territorial Units of Italy (NUTS: IT).

A total of 210 RNs were interviewed and divided into five different geographic areas, according to the Nomenclature of Statistical Territorial Units of Italy (NUTS: IT, level 1) (https://ec.europa.eu/eurostat/web/nuts/nuts-maps) (accessed on 20 May 2022). [26] (Figure 1).

The rate of female RNs was significantly higher in the Northwest and Northeast compared to the Island areas (Northwest 34/43 (79.0%) vs. Islands 7/15 (46.6%), *p*-value 0.040; Northeast 32/40 (80%) vs. Islands 7/15 (46.6%), *p*-value 0.036). No difference was found in the demographic characteristics and educational qualifications (Table 1).

The rate of ward nurses was lower in the North compared to the South and the Island areas (Northwest 21/43 (48.8%) vs. South 48/63 (76.1%), *p*-value 0.007; Northwest 21/43 (48.8%) vs. Islands 13/15 (86.6%), *p*-value 0.024; Northeast 19/40 (47.5%) vs. South 48/63 (76.1%), *p*-value 0.005; Northeast 19/40 (47.5%) vs. Islands 13/15 (86.6%), *p*-value 0.020) (Table 1).

For each query of the questionnaire, a Tuckey test and sample test for the equality of proportions were used to analyze continuous and categorical variables, respectively, in every possible comparison. With five groups, there are 10 possible comparisons (Midst vs. Islands, Northeast vs. Islands, Northwest vs. Islands, South vs. Islands, Northeast vs. Midst, Northwest vs. Midst, South vs. Midst, Northwest vs. Northeast, South vs. Northeast, South–Northwest). In Table 2 we only summarize comparisons that have achieved a significant difference. In particular, Northwest RNs receive a better education about wound care area during university courses: the rate of Northwest RNs that did not receive any training (0 h) in the wound care area during university courses was 4.6%, significantly lower than other Italian geographical areas (Northwest 2/43 (4.6%) vs. Northeast 10/40 (25%), *p*-value 0.020; Northwest 2/43 (4.6%) vs. Midst 14/49 (28.5%), *p*-value 0.006; Northwest 2/43 (4.6%) vs. Islands 5/15 (33.3%), *p*-value 0.013) (Table 2).

South RNs presented a better knowledge about factors that expose the wound to becoming chronic, care and remove wound drains, and ability to assess and examine diabetic foot, compared to the Island, Northeast, and Midst areas, respectively (Query 1.7, South 4.7 ± 1.8 vs. Islands 3.1 ± 1.9, *p*-value 0.033; Query 2.3, South 5.1 ± 2.0 vs. Islands 3.9 ± 2.2, *p*-value 0.042; Query 2.16, South 5.0 ± 1.7 vs. Midst 3.8 ± 1.9, *p*-value 0.013) (Table 2).

No other differences were found in the four competences examined areas (Table 3, Table 4, Table 5, Table 6 and Table 7).

## 4. Discussion

Wound care is a specific clinical area that involves multidisciplinary teamwork in which RNs play a pivotal role. Wounds are usually classified as either acute or chronic, and especially the latter is a significant concern for healthcare services and affected individuals and are also responsible for elevated costs for health systems and society and for decreased quality of life [16,17]. Therefore, wound care is an important area of clinical practice for nursing and, as so, academic education and teaching on wound care, both in basic nursing and in postgraduate knowledge, are fundamental in developing adequate and satisfactory competence and specific skills that are directly related to improving professional standards, patient safety, and more in general, quality of care in this area [7,13,16,17].

The aim of this study was to explore, among Italian RNs, wound care education received during basic or postgraduate nursing academic education, and several competence areas and specific wound care knowledge required to have adequate wound care nursing competence.

The hours of training received during academic training were adequate (≥9 h of specific training) in 33% of cases, whereas in 47.6% it is considered suboptimal (≤8 h), and it is totally inadequate in 19% of cases (lack of training) (Table 3).

Although 43.3% of participants achieved only a Bachelor’s degree, and only 5.7% achieved a Master’s degree (Table 1), the general self-perception in terms of the knowledge of the wound care area is quite sufficient, oscillating around the values of at least 4, considering the Likert values used with a scale from 1 to 7. Nevertheless, one must consider the possibility that there have been phenomena of social desirability and socially desirable responding (SDR) [27] that have led to the overestimation of the perceived training. This hypothesis is supported by the data available, since 47.6% of respondents received almost scarce training in wound care (between 1–8 h), and even 19% of respondents did not receive any university training in this regard (Table 3).

Moreover, with regard to the correlation between training time effectively carried out and perceived competence, it is possible to detect how, in the areas of more advanced competence in wound care, the respondents were found to be below the general trend, and in particular, in recipient and donor skin transplantation (on average 3.3 ± 2.2), in the first aid of frostbite ulcers (3.3 ± 1.9), and in the assistance of atypical wounds (3.6 ± 2.1) (Table 5). In fact, analyzing skills in these more specialized areas, the lack of adequate training time, with consequently less professional expertise, is more explicit.

Competence areas in wound care are strongly related to theoretical issues and practical training, during the academic attendance of nursing students, to develop and achieve appropriate skills to deliver optimal wound care to patients [16,17].

This study clearly showed that caring for patients with wounds requires several skills and attitudes from RNs. Moreover, wound care education in undergraduate nursing students is not always adequate and lacks consistent learning goals, content, and duration of academic training.

This study has some limitations: the findings are observational due to the cross-sectional structure of the study design; the online web-based survey may lead to selection bias and limit the generalizability of the study; the assessment of competence areas was based on self-reporting, and the participants may perceive in different ways the items that were investigated. Moreover, we were able only to assess Italian nurses’ perception of self-efficacy and of their own capacity to deal with the wound care area. Probably further studies with psychometric testing [28] may validate our results in terms of comprehensive competence assessments in the wound care area.

## 5. Conclusions

This study shows that in Italy, education in wound care in nursing students is relatively poor and, probably, many skills are achieved during RNs’ careers in an empirical way. Further research is needed to find out what educational interventions are needed for nursing students and for those RNs that already work in an area where wound care management is required in clinical practice.

## Figures and Tables

**Table 1:** Population data.

	Overall (N = 210)	Northwest (n = 43/210)	Northeast (n = 40/210)	Midst (n = 49/210)	South (n = 63/210)	Islands (n = 15/210)	*p*-Value (<0.05)
**Demographic characteristics**							
Age (years, mean ± sd)	39.3 ± 8.9	40.2 ± 8.7	38.2 ± 8.2	39.6 ± 9.5	39.4 ± 9.2	38.2 ± 8.7	0.856
Females	148/210 (70.4%)	34/43 (79.0%)	32/40 (80%)	36/49 (73.4%)	39/63 (61.9%)	7/15 (46.6%)	0.044 *
**Northwest vs. Islands**	-	34/43 (79.0%)	-	-	-	7/15 (46.6%)	0.040 *
**Northeast vs. Islands**	-	-	32/40 (80%)	-	-	7/15 (46.6%)	0.036 *
**Educational qualification**							
Bachelor’s degree in Nursing	91/210 (43.3%)	16/43 (37.2%)	19/40 (47.5%)	23/49 (46.9%)	25/63 (39.6%)	8/15 (53.3%)	0.707
Master’s degree in Nursing and Midwifery Sciences	12/210 (5.7%)	2/43 (4.6%)	1/40 (2.5%)	2/49 (4.1%)	7/63 (11.1%)	0/15 (0%)	0.247
Postgraduate diploma in wound care	12/210 (5.7%)	4/43 (9.3%)	4/40 (10%)	4/49 (8.1%)	0/63 (0%)	0/15 (0%)	0.104
Other Master’s degree	11/210 (5.2%)	6/43 (13.9%)	2/40 (5%)	0/49 (0%)	2/63 (3.1%)	1/15 (6.6%)	0.042
Bachelor’s degree in Nursing + Postgraduate diploma in wound care	5/210 (2.3%)	2/43 (4.6%)	1/40 (2.5%)	1/49 (2%)	1/63 (1.5%)	0/15 (0%)	0.823
Bachelor’s degree in Nursing + Other Master’s degree	51/210 (24.2%)	9/43 (20.9%)	9/40 (22.5%)	11/49 (22.4%)	18/63 (28.5%)	4/15 (26.6%)	0.894
Master’s degree in Nursing and Midwifery Sciences + Postgraduate diploma in wound care	3/210 (1.4%)	0/43 (0%)	0/40 (0%)	0/49 (0%)	2/63 (3.1%)	1/15 (6.6%)	0.184
Master’s degree in Nursing and Midwifery Sciences + Other Master’s degree	8/210 (3.8%)	1/43 (2.3%)	0/40 (0%)	1/49 (2%)	5/63 (7.9%)	1/15 (6.6%)	0.237
Postgraduate diploma in wound care + Other Master’s degree	2/210 (0.9%)	0/43 (0%)	0/40 (0%)	2/49 (4.1%)	0/63 (0%)	0/15 (0%)	0.156
Bachelor’s degree in Nursing + Postgraduate diploma in wound care + Other Master’s degree	6/210 (2.8%)	1/43 (2.3%)	3/40 (7.6%)	0/49 (0%)	2/63 (3.1%)	0/15 (0%)	0.281
Master’s degree in Nursing and Midwifery Sciences + Postgraduate diploma in wound care + Other Master’s degree	3/210 (1.4%)	0/43 (0%)	0/40 (0%)	2/49 (4.1%)	1/63 (1.5%)	0/15 (0%)	0.422
**Current work**							
Ward nurse	128/210 (60.9%)	21/43 (48.8%)	19/40 (47.5%)	27/49 (55.1%)	48/63 (76.1%)	13/15 (86.6%)	0.002 *
**Northwest vs. South**	-	21/43 (48.8%)	-	-	48/63 (76.1%)	-	0.007 *
**Northwest vs. Islands**	-	21/43 (48.8%)	-	-	-	13/15 (86.6%)	0.024 *
**Northeast vs. South**	-	-	19/40 (47.5%)	-	48/63 (76.1%)	-	0.005 *
**Northeast vs. Islands**	-	-	19/40 (47.5%)	-	-	13/15 (86.6%)	0.020 *
Home nurse	25/210 (11.9%)	5/43 (11.6%)	8/40 (20%)	7/49 (14.2%)	3/63 (4.7%)	2/15 (13.3%)	0.209
Private nurse	20/210 (9.5%)	6/43 (13.9%)	5/40 (12.5%)	7/49 (14.2%)	2/63 (3.1%)	0/15 (0%)	0.125
Other	37/210 (17.6%)	11/43 (25.5%)	8/40 (20%)	8/49 (16.3%)	10/63 (15.8%)	0/15 (0%)	0.245

* = statistical significance.

**Table 2:** Summary of significant comparisons.

	Overall (N = 210)	Northwest (n = 43/210)	Northeast (n = 40/210)	Midst (n = 49/210)	South (n = 63/210)	Islands (n = 15/210)	*p*-Value (<0.05)
**Significant queries**							
*Answers: 0 h, 1–8 h, 9–24 h, 24–48 h, >48 h*							
How many hours of training did you receive in the wound care area during your university training courses? (a) 0 (h)	40/210 (19%)	2/43 (4.6%)	10/40 (25%)	14/49 (28.5%)	9/63 (14.2%)	5/15 (33.3%)	0.014 *
**Northwest vs. Northeast**	-	2/43 (4.6%)	10/40 (25%)	-	-	-	0.020 *
**Northwest vs. Midst**	-	2/43 (4.6%)	-	14/49 (28.5%)	-	-	0.006 *
**Northwest vs. Islands**	-	2/43 (4.6%)	-	-	-	5/15 (33.3%)	0.013 *
*Answers: strongly disagree 1–2–3–4–5–6–7-strongly agree*							
*1.7 Your level of knowledge about factors that expose the wound to becoming chronic is adequate?* (mean ± sd)	4.2 ± 2.0	4.0 ± 2.0	4.2 ± 2.1	4.2 ± 1.9	4.7 ± 1.8	3.1 ± 1.9	0.052 *
**South vs. Islands**	-	-	-	-	4.7 ± 1.8	3.1 ± 1.9	0.033 *
*2.3 I can care and remove wound drains* (mean ± sd)	4.6 ± 2.1	4.5 ± 2.2	3.9 ± 2.2	4.5 ± 2.0	5.1 ± 2.0	4.8 ± 2.2	0.078 *
**South vs. Northeast**	-	-	3.9 ± 2.2	-	5.1 ± 2.0	-	0.042 *
*2.16 I can assess and examine diabetic foot* (mean ± sd)	4.3 ± 1.9	4.3 ± 1.9	4.2 ± 2.0	3.8 ± 1.9	5.0 ± 1.7	3.7 ± 1.5	0.013 *
**South vs. Midst**	-	-	-	3.8 ± 1.9	5.0 ± 1.7	-	0.013 *

* = statistical significance.

**Table 3:** Educational wound care area. Each query expects a categorical answer between five different ranges of hours (0 h, 1–8 h, 9–24 h, 24–48 h, or more than 48 h).

	Overall (N = 210)	Northwest (n = 43/210)	Northeast (n = 40/210)	Midst (n = 49/210)	South (n = 63/210)	Islands (n = 15/210)	*p*-Value (<0.05)
**Educational wound care area**							
*Answers: 0 h, 1–8 h, 9–24 h, 24–48 h, >48 h*							
*How many hours of training did you receive in the wound care area during your university training courses?*							
(a) 0 (h)	40/210 (19%)	2/43 (4.6%)	10/40 (25%)	14/49 (28.5%)	9/63 (14.2%)	5/15 (33.3%)	0.014 *
(b) 1–8 (h)	100/210 (47.6%)	22/43 (51.1%)	15/40 (37.5%)	25/49 (51%)	31/63 (49.2%)	7/15 (46.6%)	0.707
(c) 9–24 (h)	21/210 (10%)	5/43 (11.6%)	4/40 (10%)	2/49 (4.1%)	9/63 (14.2%)	1/15 (6.6%)	0.477
(d) 24–48 (h)	11/210 (5.2%)	2/43 (4.6%)	2/40 (5%)	1/49 (2%)	6/63 (9.5%)	0/15 (0%)	0.379
(e) >48 (h)	38/210 (18.1%)	12/43 (27.9%)	9/40 (22.5%)	7/49 (14.2%)	8/63 (12.6%)	2/15 (13.3%)	0.261

* = statistical significance.

**Table 4:** Competence area in wound care: anatomy and physiology area. Each query expects a score value as an answer, based on a seven-point scale (Likert scale) ranging from 1 (strongly disagree) to 7 (strongly agree). Results are shown as mean ± standard deviation (mean ± sd).

	Overall (N = 210)	Northwest (n = 43/210)	Northeast (n = 40/210)	Midst (n = 49/210)	South (n = 63/210)	Islands (n = 15/210)	*p*-Value (<0.05)
**Competence area in wound care**							
**1.** **Anatomy and Physiology area**							
*Answers: strongly disagree 1–2–3–4–5–6–7-strongly agree*							
*1.1 Your level of knowledge in anatomy and physiology of skin and tissues is adequate?* (mean ± sd)	4.2 ± 1.8	4.2 ± 1.9	4.3 ± 1.8	4.1 ± 1.8	4.5 ± 1.8	3.2 ± 1.8	0.188
*1.2 Your level of knowledge about symptoms and findings of peripheral artery disease is adequate?* (mean ± sd)	4.0 ± 1.9	4.2 ± 1.9	3.9 ± 1.9	3.8 ± 1.8	4.4 ± 2.0	3.2 ± 1.8	0.180
*1.3 Your level of knowledge about symptoms and findings of venous insufficiency of lower limbs is adequate?* (mean ± sd)	4.0 ± 2.0	4.2 ± 2.0	4.0 ± 2.1	3.8 ± 1.9	4.3 ± 2.0	3.1 ± 1.9	0.251
*1.4 Your level of knowledge about factors that regulate wound healing is adequate?* (mean ± sd)	4.5 ± 1.9	4.6 ± 1.8	4.5 ± 2.0	4.6 ± 1.9	4.7 ± 1.8	3.4 ± 2.1	0.187
*1.5 Your level of knowledge about factors that affect wound healing is adequate?* (mean ± sd)	4.5 ± 1.9	4.5 ± 1.9	4.4 ± 2.0	4.6 ± 1.9	4.7 ± 1.8	3.4 ± 2.2	0.230
*1.6 Your level of knowledge about wound healing phases is adequate?* (mean ± sd)	4.6 ± 1.9	4.5 ± 1.8	4.5 ± 2.0	5.0 ± 1.8	4.8 ± 1.8	3.6 ± 2.1	0.121
*1.7 Your level of knowledge about factors that expose the wound to becoming chronic is adequate?* (mean ± sd)	4.2 ± 2.0	4.0 ± 2.0	4.2 ± 2.1	4.2 ± 1.9	4.7 ± 1.8	3.1 ± 1.9	0.052 *

* = statistical significance.

**Table 5:** Competence area in wound care: care of chronic and acute wounds. Each query expects a score value as an answer, based on a seven-point scale (Likert scale) ranging from 1 (strongly disagree) to 7 (strongly agree). Results are shown as mean ± standard deviation (mean ± sd).

	Overall (N = 210)	Northwest (n = 43/210)	Northeast (n = 40/210)	Midst (n = 49/210)	South (n = 63/210)	Islands (n = 15/210)	*p*-Value (<0.05)
**2.** **Competence area in wound care**							
**Care of chronic and acute wounds**							
*Answers: strongly disagree 1–2–3–4–5–6–7-strongly agree*							
*2.1 I can assess a surgical wound by means of sterile and clean techniques* (mean ± sd)	5.4 ± 1.8	5.5 ± 1.9	5.3 ± 1.9	5.4 ± 1.6	5.5 ± 1.6	4.6 ± 2.0	0.981
*2.2 I can assess the most common complication of a surgical wound* (mean ± sd)	5.0 ± 1.8	5.1 ± 2.0	4.8 ± 1.9	5.0 ± 1.7	5.3 ± 1.7	4.1 ± 1.9	0.240
*2.3 I can care and remove wound drains* (mean ± sd)	4.6 ± 2.1	4.5 ± 2.2	3.9 ± 2.2	4.5 ± 2.0	5.1 ± 2.0	4.8 ± 2.2	0.078 *
*2.4 I can remove sutures/staples* (mean ± sd)	5.5 ± 1.9	5.7 ± 1.9	5.4 ± 2.1	5.7 ± 1.8	5.6 ± 1.8	4.6 ± 2.2	0.406
*2.5 I can care recipient site in skin transplantation* (mean ± sd)	3.3 ± 2.2	3.1 ± 2.3	3.6 ± 2.3	3.2 ± 2.2	3.5 ± 2.0	2.8 ± 1.8	0.662
*2.6 I can care recipient site in skin transplantation* (mean ± sd)	3.3 ± 2.2	3.1 ± 2.4	3.5 ± 2.3	3.3 ± 2.3	3.4 ± 2.0	2.6 ± 1.7	0.644
*2.7 I can provide first aid in traumatic wounds* (mean ± sd)	4.9 ± 1.8	4.9 ± 2.0	4.8 ± 1.7	4.9 ± 1.9	5.0 ± 1.8	4.3 ± 1.9	0.767
*2.8 I can assess and care traumatic wounds* (mean ± sd)	4.8 ± 1.9	4.8 ± 2.0	4.8 ± 1.9	4.7 ± 1.9	5.0 ± 1.8	4.2 ± 1.8	0.617
*2.9 I can provide first aid in burn injuries* (mean ± sd)	4.6 ± 1.8	4.4 ± 1.9	4.4 ± 1.7	4.8 ± 1.8	4.9 ± 1.8	4.6 ± 1.6	0.615
*2.10 I can assess (degree and size) and care burn injuries* (mean ± sd)	4.5 ± 1.9	4.2 ± 2.0	4.2 ± 1.9	4.8 ± 1.8	4.6 ± 1.8	4.2 ± 1.8	0.468
*2.11 I can provide first aid in frostbite* (mean ± sd)	3.4 ± 2.0	2.9 ± 1.9	3.3 ± 1.9	3.5 ± 1.9	3.9 ± 2.0	2.8 ± 1.6	0.064
*2.12 I can assess (degree and size) and care in frostbite* (mean ± sd)	3.3 ± 1.9	2.9 ± 1.9	3.1 ± 1.9	3.4 ± 1.9	3.9 ± 2.0	2.8 ± 1.8	0.076
*2.13 I can assess oedema* (mean ± sd)	4.9 ± 1.8	4.8 ± 1.9	4.7 ± 2.0	5.3 ± 1.9	5.0 ± 1.7	4.0 ± 1.7	0.214
*2.14 I can correctly apply compression therapy* (mean ± sd)	4.1 ± 2.1	3.9 ± 2.1	3.9 ± 2.3	4.0 ± 2.3	4.6 ± 2.0	3.5 ± 1.7	0.246
*2.15 I can assess arterial circulation* (mean ± sd)	4.1 ± 2.2	4.1 ± 2.0	3.9 ± 2.2	3.7 ± 2.4	4.7 ± 2.2	3.3 ± 1.9	0.107
*2.16 I can assess and examine diabetic foot* (mean ± sd)	4.3 ± 1.9	4.3 ± 1.9	4.2 ± 2.0	3.8 ± 1.9	5.0 ± 1.7	3.7 ± 1.5	0.013 *
*2.17 I am skilled in principles of offloading in the management of diabetic foot* (mean ± sd)	4.0 ± 1.9	4.0 ± 1.9	3.9 ± 2.1	3.6 ± 2.0	4.6 ± 1.8	3.7 ± 1.4	0.124
*2.18 I can assess risk factors for pressure ulcer/injury* (mean ± sd)	5.1 ± 1.8	5.2 ± 1.7	5.1 ± 1.8	5.2 ± 2.0	5.1 ± 1.7	4.4 ± 1.8	0.675
*2.19 I am skilled in pressure and friction relief in pressure ulcer/injury* (mean ± sd)	5.1 ± 1.9	5. 2 ± 1.8	5.1 ± 1.9	5.3 ± 2.0	5.0 ± 1.8	4.4 ± 1.8	0.618
*2.20 I can assess risk factors for skin tears* (mean ± sd)	4.1 ± 2.3	4.3 ± 2.4	4.3 ± 2.4	4.2 ± 2.2	4.2 ± 2.1	3.0 ± 2.3	0.370
*2.21 I am skilled in skin care and protection in skin tears* (mean ± sd)	4.1 ± 2.3	4.1 ± 2.4	4.2 ± 2.4	4.2 ± 2.3	4.1 ± 2.1	3.1 ± 2.5	0.548
*2.22 I can assess atypical wounds* (mean ± sd)	3.6 ± 2.1	3.5 ± 2.0	3.5 ± 2.3	3.2 ± 2.1	4.1 ± 2.0	2.9 ± 2.0	0.165

* = statistical significance.

**Table 6:** Competence area in wound care: wound management and care of a patent with a wound. Each query expects a score value as an answer, based on a seven-point scale (Likert scale) ranging from 1 (strongly disagree) to 7 (strongly agree). Results are shown as mean ± standard deviation (mean ± sd).

	Overall (N = 210)	Northwest (n = 43/210)	Northeast (n = 40/210)	Midst (n = 49/210)	South (n = 63/210)	Islands (n = 15/210)	*p*-Value (<0.05)
*Answers: strongly disagree 1–2–3–4–5–6–7-strongly agree*							
**Wound management and care of a patent with a wound**							
*3.1 I know the principles of working aseptically in wound management (procedure preparation, correct use of personal protective equipment, aseptic working and related order)* (mean ± sd)	5.3 ± 1.6	5.5 ± 1.6	5.3 ± 1.7	5.1 ± 1.6	5.3 ± 1.7	4.8 ± 1.7	0.587
*3.2 I know the principles of assessing and care of an open wound and I can assess the wound bed (colour and tissue types, environment for wound healing, evaluation of the skin surrounding the wound)* (mean ± sd)	5.1 ± 1.7	5.3 ± 1.7	5.3 ± 1.9	5.2 ± 1.7	5.0 ± 1.6	4.6 ± 1.6	0.684
*3.3 I know the principles of assessment and care of an infected wound (signs and classification of an infection, bacterial sample, care of an infected wound)* (mean ± sd)	5.0 ± 1.7	5.1 ± 1.8	5.1 ± 1.9	4.9 ± 1.7	5.0 ± 1.7	4.5 ± 1.7	0.836
*3.4 I know and I can use different wound debridement methods, products, and instruments* (mean ± sd)	4.8 ± 1.9	4.9 ± 1.8	4.7 ± 2.1	4.9 ± 2.0	4.7 ± 1.9	4.2 ± 1.8	0.724
*3.5 I know and I can use different wound care products and their functions, and can use products of each group correctly* (mean ± sd)	4.7 ± 1.9	4.6 ± 1.9	4.9 ± 2.0	4.9 ± 1.9	4.7 ± 1.9	4.2 ± 1.7	0.741
*3.6 I understand the importance of nutrition in wound prevention and healing and can assess patient’s nutrition status* (mean ± sd)	5.6 ± 1.5	5.5 ± 1.7	5.7 ± 1.6	5.9 ± 1.8	5.5 ± 1.4	5.4 ± 1.8	0.509
*3.7 I can assess and manage wound related pain* (mean ± sd)	4.9 ± 1.8	4.8 ± 1.8	5.1 ± 2.0	5.1 ± 1.7	5.0 ± 1.7	4.3 ± 2.2	0.577
*3.8 I can document the description and management of the wound and make a care plan* (mean ± sd)	4.8 ± 2.0	4.8 ± 2.0	4.7 ± 2.2	5.1 ± 1.9	4.7 ± 1.9	4.7 ± 2.0	0.777
*3.9 I can educate and motivate the patient with a wound (informing the patient and next of kin and stimulate self-care)* (mean ± sd)	5.2 ± 1.8	5.0 ± 1.8	5.3 ± 1.9	5.3 ± 1.9	5.1 ± 1.9	5.3 ± 1.5	0.958

**Table 7:** Values and attitudes. Each query expects a score value as an answer, based on a seven-point scale (Likert scale) ranging from 1 (strongly disagree) to 7 (strongly agree). Results are shown as mean ± standard deviation (mean ± sd).

	Overall (N = 210)	Northwest (n = 43/210)	Northeast (n = 40/210)	Midst (n = 49/210)	South (n = 63/210)	Islands (n = 15/210)	*p*-Value (<0.05)
**Values and attitudes**							
*Answers: strongly disagree 1–2–3–4–5–6–7-strongly agree*							
*4.1 I understand the importance of multi-professional working and consultations when caring for a patient with a wound* (mean ± sd)	6.3 ± 1.0	6.1 ± 1.4	6.7 ± 0.7	6.2 ± 1.2	6.3 ± 0.9	6.4 ± 0.6	0.164
*4.2 I understand the meaning of holistic and patient-centered care when caring for a patient with a wound* (mean ± sd)	6.1 ± 1.2	6.0 ± 1.3	6.4 ± 1.0	6.0 ± 1.5	6.1 ± 1.2	6.4 ± 0.6	0.581
*4.3 I understand the importance to respect patient’s privacy and autonomy in wound care* (mean ± sd)	6.3 ± 1.0	6.4 ± 1.0	6.5 ± 0.8	6.2 ± 1.0	6.2 ± 1.2	6.6 ± 0.6	0.473
*4.4 I understand the importance to act professionally when caring for wounds* (mean ± sd)	6.4 ± 1.0	6.4 ± 0.9	6.5 ± 0.9	6.2 ± 1.2	6.4 ± 0.9	6.6 ± 0.6	0.626
*4.5 I understand the importance of economic perspectives of care from the patient’s and society’s point of view and I am fully aware of wound care costs* (mean ± sd)	6.3 ± 1.1	6.2 ± 1.1	6.4 ± 1.2	6.3 ± 1.1	6.3 ± 1.1	6.5 ± 0.7	0.867

## Data Availability

Datasets are available on request from the corresponding author only as the data are sensitive, and participants may be potentially identifiable.

## Supplementary Materials

The following supporting information can be downloaded at: https://www.mdpi.com/article/10.3390/nursrep12030067/s1, Supplementary material S1, List of Italian universities providing Bachelor's degree in Nursing; Supplementary material S2, List of Italian universities providing master's degree in nursing and midwifery sciences; Supplementary material S3, List of Italian universities providing Master's degree in Wound Care; Supplementary material S4, Questionnaire.

## References

[1] Martinengo L., Olsson M., Bajpai R., Soljak M., Upton Z., Schmidtchen A., Car J., Järbrink K. (2019). Prevalence of chronic wounds in the general population: Systematic review and meta-analysis of observational studies. Ann. Epidemiol..

[2] Costa D., Ielapi N., Caprino F., Giannotta N., Sisinni A., Abramo A., Ssempijja L., Andreucci M., Bracale U.M., Serra R. (2022). Social Aspects of Diabetic Foot: A Scoping Review. Soc. Sci..

[3] McCann C., Watson A., Barnes D. (2022). Major burns: Part 1. Epidemiology, pathophysiology and initial management. BJA Educ..

[4] Serra R., Barbetta A., Ielapi N., De Franciscis S., Gasbarro V. (2017). Current knowledge on venous and lymphatic ulcers. A systematic review on evidence-based medicine. Acta Phlebol..

[5] Serra R., Butrico L., Ruggiero M., Rossi A., Buffone G., Fugetto F., De Caridi G., Massara M., Falasconi C., Rizzuto A. (2015). Epidemiology, diagnosis and treatment of chronic leg ulcers: A systematic review. Acta Phlebol..

[6] Serra R., Ielapi N., Barbetta A., de Franciscis S. (2018). Skin tears and risk factors assessment: A systematic review on evidence-based medicine. Int. Wound J..

[7] Sürme Y., Kartın P.T., Çürük G.N. (2018). Knowledge and Practices of Nurses Regarding Wound Healing. J. Perianesthesia Nurs. Off. J. Am. Soc. PeriAnesthesia Nurses.

[8] Yao K., Bae L., Yew W.P. (2013). Post-operative wound management. Aust. Fam. Physician.

[9] Özaydın İ., Özaydın Ç. (2010). Surgical site infections. J. Konuralp Med..

[10] Jeschke M.G., van Baar M.E., Choudhry M.A., Chung K.K., Gibran N.S., Logsetty S. (2020). Burn injury. Nat. Rev. Dis. Primers.

[11] Leaper D.J. (2006). Traumatic and surgical wounds. BMJ Clin. Res. Ed..

[12] Nygaard R.M., Endorf F.W. (2018). Frostbite vs. Burns: Increased Cost of Care and Use of Hospital Resources. J. Burn. Care Res. Off. Publ. Am. Burn. Assoc..

[13] Kielo-Viljamaa E., Suhonen R., Jalonen L., Stolt M. (2022). Areas of nursing competence in acute wound care: A focus group study. Collegian.

[14] Kielo-Viljamaa E., Viljamaa J., Suhonen R., Salminen L., Stolt M. (2022). Learning goals and content for wound care education in Finnish nursing education—A Delphi study. Nurse Educ. Today.

[15] Kielo E., Salminen L., Stolt M. (2018). Graduating student nurses’ and student podiatrists’ wound care competence—An integrative literature review. Nurse Educ. Pract..

[16] Kielo E., Salminen L., Suhonen R., Puukka P., Stolt M. (2019). Graduating student nurses’ and podiatrists’ theoretical wound care competence a cross-sectional study. J. Wound Care.

[17] Kielo E., Suhonen R., Salminen L., Stolt M. (2019). Competence areas for registered nurses and podiatrists in chronic wound care, and their role in wound care practice. J. Clin. Nurs..

[18] Welsh L. (2018). Wound care evidence, knowledge and education amongst nurses: A semi-systematic literature review. Int. Wound J..

[19] Palese A., Zabalegui A., Sigurdardottir A.K., Bergin M., Dobrowolska B., Gasser C., Pajnkihar M., Jackson C. (2014). Bologna process, more or less: Nursing education in the European economic area: A discussion paper. Int. J. Nurs. Educ. Scholarsh..

[20] Marchetti A., Venturini G., Virgolesi M., Gobbi M., Rocco G., Pulimeno A.M.L., Stievano A., Piredda M., De Marinis M.G. (2015). Tuning nursing educational in an Italian academic context. Nurse Educ. Today.

[21] Universitaly. https://www.universitaly.it/index.php/cercacorsi/universita.

[22] Almalaurea. https://www.almalaurea.it/lau/postlaurea/aa2019-2020.

[23] Strobe Statement Strenghtening the Reporting of Observational Studies in Epidemiology. https://www.strobe-statement.org/index.php?id=strobe-home.

[24] Ielapi N., Andreucci M., Bracale U.M., Costa D., Bevacqua E., Bitonti A., Mellace S., Buffone G., Candido S., Provenzano M. (2021). Insomnia Prevalence among Italian Night-Shift Nurses. Nurs. Rep..

[25] Ielapi N., Andreucci M., Bracale U.M., Costa D., Bevacqua E., Giannotta N., Mellace S., Buffone G., Cerabona V., Arturi F. (2021). Workplace Violence towards Healthcare Workers: An Italian Cross-Sectional Survey. Nurs. Rep..

[26] Nuts—Nomenclature of Territorial Units for Statistics. https://ec.europa.eu/eurostat/web/nuts/nuts-maps.

[27] Latkin C.A., Edwards C., Davey-Rothwell M.A., Tobin K.E. (2017). The relationship between social desirability bias and self-reports of health, substance use, and social network factors among urban substance users in Baltimore, Maryland. Addict. Behav..

[28] Lichtenberg J., Portnoy S.M., Bebeau M.J., Leigh I.W., Nelson P.D., Rubin N.J., Smith I.L., Kaslow N. (2007). Challenges to the Assessment of Competence and Competencies. Prof. Psychol. Res. Pract..

