# Supplementary material for: Wound Care Self-Efficacy Assessment of Italian Registered Nurses and Wound Care Education in Italian Nursing Education System: A Cross-Sectional Study
Source: Nurs Rep. 2022 Sep 18;12(3):674–84. doi: 10.3390/nursrep12030067 (PMC9506369; doi:10.3390/nursrep12030067)
Supplement: Supplementary file 1 [file nursrep-12-00067-s001.zip › nursrep-1894396-supplementary.pdf]

SUPPLEMENTARY MATERIAL S1

Supplementary material S1. List of Italian universities providing Bachelor's degree in Nursing.

**University**	**Region**
University Aldo moro	Puglia
University of Bologna	Emilia Romagna
University of Brescia	Lombardy
University of Cagliari	Sardinia
University of Catania	Sicily
University Magna Graecia of Catanzaro	Calabria
University Gabriele D'annunzio Chieti-Pescara	Abruzzo
University of Ferrara	Emilia Romagna
University of Firenze	Tuscany
University of Foggia	Puglia
University of Genova	Liguria
University Insubria of Varese-Como	Lombardy
University of Aquila	Abruzzo
University of Messina	Sicily
University of Milano	Lombardy
University of Milano-Bicocca	Lombardy
University Sacro Cuore of Milano	Lombardy
University Humanitas	Lombardy
University San Raffaele-Milano	Lombardy
University of Modena and Reggio Emilia	Emilia Romagna
University of Molise	Molise
University Federico II-Napoli	Campania
University Luigi Vanvitelli	Campania
University of Padova	Veneto
University of Palermo	Sicily
University of Parma	Emilia Romagna
University of Pavia	Lombardy
University of Perugia	Umbria
University of Piemonte orientale, "Amedeo Avogadro"-Vercelli	Piedmont
University of Pisa	Tuscany
University Politecnica of Marche	Marche
University La Sapienza-Rome	Lazio
University Tor Vergata-Rome	Lazio
University Campus Biomedico-Roma	Lazio
University Unicamillus - Saint Camillus International University of Health Sciences	Lazio

University of Salerno	Campania
University of Sassari	Sardinia
University of Siena	Tuscany
University of Torino	Piedmont
University of Trieste	Friuli-Venezia-Giulia
University of Udine	Friuli-Venezia-Giulia
University of Verona	Veneto
**Total**	42

SUPPLEMENTARY MATERIAL S2

Supplementary material S2. List of Italian universities providing master's degree in nursing and midwifery sciences

**University**	**Region**
University Aldo moro	Puglia
University of Bologna	Emilia Romagna
University of Brescia	Lombardy
University of Catania	Sicily
University Magna Graecia of Catanzaro	Calabria
University Gabriele D'annunzio Chieti-Pescara	Abruzzo
University of Ferrara	Emilia Romagna
University of Firenze	Tuscany
University of Genova	Liguria
University of Aquila	Abruzzo
University of Messina	Sicily
University of Milano	Lombardy
University of Milano-Bicocca	Lombardy
University Sacro Cuore of Milano	Lombardy
University Humanitas	Lombardy
University San Raffaele-Milano	Lombardy
University of Modena and Reggio Emilia	Emilia Romagna
University of Molise	Molise
University Federico II-Napoli	Campania
University Luigi Vanvitelli	Campania
University of Padova	Veneto
University of Palermo	Sicily
University of Parma	Emilia Romagna
University of Pavia	Lombardy
University of Perugia	Umbria
University of Piemonte orientale, "Amedeo Avogadro"-Vercelli	Piedmont

University of Pisa	Tuscany
University Politecnica of Marche	Marche
University La Sapienza-Rome	Lazio
University Tor Vergata-Rome	Lazio
University of Siena	Tuscany
University of Torino	Piedmont
University of Trieste	Friuli-Venezia-Giulia
University of Verona	Veneto
**Total**	34

SUPPLEMENTARY MATERIAL S3

Supplementary material S3. List of Italian universities providing Master's degree in Wound Care

**University**	**Region**	**Name**	**University department**
University of Catania	Sicily	Wound care and diabetic foot	Department of General Surgery and Medical Surgical Specialties
University Magna Graecia of Catanzaro	Calabria	Wound care and management of skin injuries	Department of Medical and Surgical Sciences
University of Pavia	Lombardy	Nurse specialist in wound care- V edition	Department of Clinical Surgical, Diagnostic and Pediatric Sciences
University of Torino	Piedmont	Wound care	Department of Medical Sciences
**Total**	4		

SUPPLEMENTARY MATERIAL S4

QUESTIONNAIRE

A. ***PERSONAL INFORMATION SECTION***

FAMILY NAME

FIRST NAME

GENDER (M/F)

AGE:

EDUCATIONAL QUALIFICATION:
Bachelor's degree in Nursing
Master's degree in Nursing and Midwifery Sciences
Postgraduate diploma in Wound Care
Other postgraduate diploma ……………………..

CURRENT WORK
Ward nurse
Home nurse
Private nurse
Other …………..

B. EDUCATIONAL WOUND CARE AREA
How many hours of training did you receive in the wound care area during your university training courses?

A. *0*
B. *1-8*
C. *9-24*
D. *24-48*
E. *> 48*

C. ***COMPETENCE AREA IN WOUND CARE***

1. ANATOMY AND PHYSIOLOGY AREA
1.1 - Your level of knowledge in anatomy and physiology of skin and tissues is adequate

strongly disagree [1], [2], [3], [4], [5], [6], [7] strongly agree

1.2 - Your level of knowledge about symptoms and findings of peripheral artery disease is adequate
strongly disagree [1], [2], [3], [4], [5], [6], [7] strongly agree

1.3 - Your level of knowledge about symptoms and findings of venous insufficiency of lower limbs is adequate
strongly disagree [1], [2], [3], [4], [5], [6], [7] strongly agree

1.4 - Your level of knowledge about factors that regulate wound healing is adequate
strongly disagree [1], [2], [3], [4], [5], [6], [7] strongly agree

1.5 - Your level of knowledge about factors that affect wound healing is adequate
strongly disagree [1], [2], [3], [4], [5], [6], [7] strongly agree

1.6 - Your level of knowledge about wound healing phases is adequate
strongly disagree [1], [2], [3], [4], [5], [6], [7] strongly agree

1.7- Your level of knowledge about factors that expose the wound to becoming chronic is adequate
strongly disagree [1], [2], [3], [4], [5], [6], [7] strongly agree

2. CARE OF CHRONIC AND ACUTE WOUNDS
2.1 - I can assess a surgical wound by means of sterile and clean techniques
strongly disagree [1], [2], [3], [4], [5], [6], [7] strongly agree

2.2 - I can assess the most common complication of a surgical wound
strongly disagree [1], [2], [3], [4], [5], [6], [7] strongly agree

2.3 - I can care and remove wound drains
strongly disagree [1], [2], [3], [4], [5], [6], [7] strongly agree

2.4 - I can remove sutures / staples
strongly disagree [1], [2], [3], [4], [5], [6], [7] strongly agree

2.5 I can care recipient site in skin transplantation
strongly disagree [1], [2], [3], [4], [5], [6], [7] strongly agree

2.6 I can care donor site in skin transplantation
strongly disagree [1], [2], [3], [4], [5], [6], [7] strongly agree

2.7 I can provide first aid in traumatic wounds
strongly disagree [1], [2], [3], [4], [5], [6], [7] strongly agree

2.8 I can assess and care traumatic wounds
strongly disagree [1], [2], [3], [4], [5], [6], [7] strongly agree

2.9 I can provide first aid in burn injuries
strongly disagree [1], [2], [3], [4], [5], [6], [7] strongly agree

2.10 I can assess (degree and size) and care burn injuries
strongly disagree [1], [2], [3], [4], [5], [6], [7] strongly agree

2.11 I can provide first aid in frostbite
strongly disagree [1], [2], [3], [4], [5], [6], [7] strongly agree

2.12 I can assess (degree and size) and care in frostbite
strongly disagree [1], [2], [3], [4], [5], [6], [7] strongly agree

2.13 I can assess oedema
strongly disagree [1], [2], [3], [4], [5], [6], [7] strongly agree

2.14 I can correctly apply compression therapy
strongly disagree [1], [2], [3], [4], [5], [6], [7] strongly agree

2.15 I can assess arterial circulation
strongly disagree [1], [2], [3], [4], [5], [6], [7] strongly agree

2.16 I can assess and examine diabetic foot
strongly disagree [1], [2], [3], [4], [5], [6], [7] strongly agree

2.17 I am skilled in principles of offloading in the management of diabetic foot
strongly disagree [1], [2], [3], [4], [5], [6], [7] strongly agree

2.18 I can assess risk factors for pressure ulcer/injury
strongly disagree [1], [2], [3], [4], [5], [6], [7] strongly agree

2.19 I am skilled in pressure and friction relief in pressure ulcer/injury
strongly disagree [1], [2], [3], [4], [5], [6], [7] strongly agree

2.20 I am skilled in skin care and protection in pressure ulcer/injury
strongly disagree [1], [2], [3], [4], [5], [6], [7] strongly agree

2.21 I can assess risk factors for skin tears
strongly disagree [1], [2], [3], [4], [5], [6], [7] strongly agree

2.22 I am skilled in skin care and protection in skin tears
strongly disagree [1], [2], [3], [4], [5], [6], [7] strongly agree

2.23 – I can assess atypical wounds
strongly disagree [1], [2], [3], [4], [5], [6], [7] strongly agree

3. WOUND MANAGEMENT AND CARE OF A PATIENT WITH A WOUND

3.1 – I know the principles of working aseptically in wound management (procedure preparation, correct use of personal protective equipment, aseptic working and related order)

strongly disagree [1], [2], [3], [4], [5], [6], [7] strongly agree.

3.2 I know the principles of assessing and care of an open wound and I can assess the wound bed (colour and tissue types, environment for wound healing, evaluation of the skin surrounding the wound)
strongly disagree [1], [2], [3], [4], [5], [6], [7] strongly agree.

3.3 I know the principles of assessment and care of an infected wound (signs and classification of an infection, bacterial sample, care of an infected wound)
strongly disagree [1], [2], [3], [4], [5], [6], [7] strongly agree.

3.4 I know and I can use different wound debridement methods, products, and instruments
strongly disagree [1], [2], [3], [4], [5], [6], [7] strongly agree.

3.5 I know and I can use different wound care products and their functions, and can use products of each group correctly
strongly disagree [1], [2], [3], [4], [5], [6], [7] strongly agree.

3.6 I understand the importance of nutrition in wound prevention and healing and can assess patient's nutrition status.
strongly disagree [1], [2], [3], [4], [5], [6], [7] strongly agree.

3.7 I can assess and manage wound related pain
strongly disagree [1], [2], [3], [4], [5], [6], [7] strongly agree.

3.8 I can document the description and management of the wound and make a care plan
strongly disagree [1], [2], [3], [4], [5], [6], [7] strongly agree.

3.9 I can educate and motivate the patient with a wound (informing the patient and next of kin and stimulate self-care)
strongly disagree [1], [2], [3], [4], [5], [6], [7] strongly agree.

4. VALUES AND ATTITUDES
4.1 I understand the importance of multi-professional working and consultations when caring for a patient with a wound.
strongly disagree [1], [2], [3], [4], [5], [6], [7] strongly agree.

4.2 I understand the meaning of holistic and patient-centered care when caring for a patient with a wound.
strongly disagree [1], [2], [3], [4], [5], [6], [7] strongly agree.

4.3 I understand the importance to respect patient's privacy and autonomy in wound care
strongly disagree [1], [2], [3], [4], [5], [6], [7] strongly agree.

4.4 I understand the importance to act professionally when caring for wounds
strongly disagree [1], [2], [3], [4], [5], [6], [7] strongly agree.

4.5 I understand the importance of economic perspectives of care from the patient's and society's point of view and I am fully aware of wound care costs.
strongly disagree [1], [2], [3], [4], [5], [6], [7] strongly agree.
